# Heterologous Expression and Auto-Activation of Human Pro-Inflammatory Caspase-1 in *Saccharomyces cerevisiae* and Comparison to Caspase-8

**DOI:** 10.3389/fimmu.2021.668602

**Published:** 2021-07-14

**Authors:** Marta Valenti, María Molina, Víctor J. Cid

**Affiliations:** Departamento de Microbiología y Parasitología, Facultad de Farmacia, Instituto Ramón y Cajal de Investigaciones Sanitarias (IRYCIS), Universidad Complutense de Madrid, Madrid, Spain

**Keywords:** yeast, humanized yeast models, heterologous expression, caspase-1, death domain

## Abstract

Caspases are a family of cysteine proteases that play an essential role in inflammation, apoptosis, cell death, and development. Here we delve into the effects caused by heterologous expression of human caspase-1 in the yeast *Saccharomyces cerevisiae* and compare them to those of caspase-8. Overexpression of both caspases in the heterologous model led to their activation and caused mitochondrial hyperpolarization, damage to different organelles, and cell death. All these effects were dependent on their protease activity, and caspase-8 was more aggressive than caspase-1. Growth arrest could be at least partially explained by dysfunction of the actin cytoskeleton as a consequence of the processing of the yeast Bni1 formin, which we identify here as a likely direct substrate of both caspases. Through the modulation of the *GAL1* promoter by using different galactose:glucose ratios in the culture medium, we have established a scenario in which caspase-1 is sufficiently expressed to become activated while yeast growth is not impaired. Finally, we used the yeast model to explore the role of death-fold domains (DD) of both caspases in their activity. Peculiarly, the DDs of either caspase showed an opposite involvement in its intrinsic activity, as the deletion of the caspase activation and recruitment domain (CARD) of caspase-1 enhanced its activity, whereas the deletion of the death effector domain (DED) of caspase-8 diminished it. We show that caspase-1 is able to efficiently process its target gasdermin D (GSDMD) when co-expressed in yeast. In sum, we propose that *S. cerevisiae* provides a manageable tool to explore caspase-1 activity and structure–function relationships.

## Introduction

**Figure 1:**
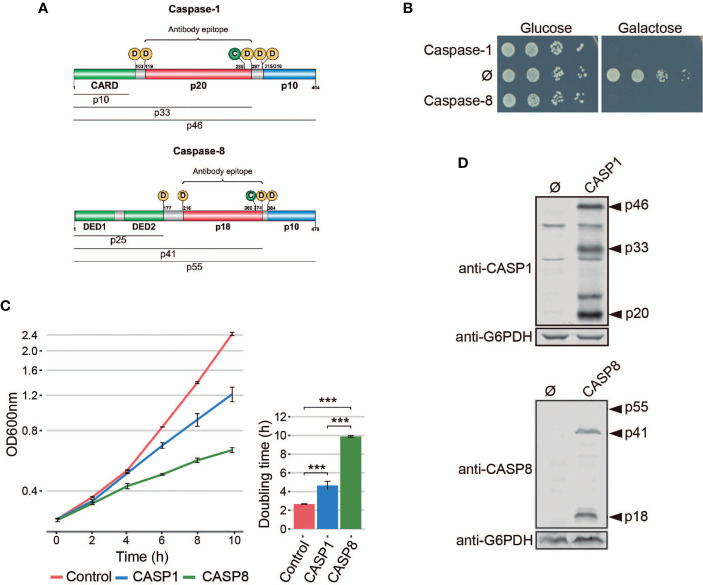
Heterologous expression of human caspase-1 and caspase-8 inhibits *S. cerevisiae* cell growth. **(A)** Schematic representation of Caspase-1 and 8 depicting their respective DDs (green), long (red), and short (blue) catalytic subunits. Their potential cleavage products and their size, the autocleavage aspartic residues (D), the cysteine residue at the catalytic center (C), and the epitopes recognized by their respective antibodies are also indicated. **(B)** Spot growth assay of BY4741 strain bearing pAG413-Caspase-1 and pAG413-Caspase-8. pAG413 empty vector (Ø) was used as a negative control. Cells were cultured on SD (Glucose) and SG (Galactose) agar media for repression and induction of caspase-1 and caspase-8 expression, respectively. A representative assay from three different experiments with different transformant clones is shown. **(C)** Growth curves of cells bearing the same plasmids as in panel **(B)** performed in SG medium. Measures of OD_600_ were taken each two hours throughout the exponential growth phase. Results are represented as OD_600_ vs time in a semilogarithmic plot (left panel). Doubling times were determined by calculating the slope over the linear portion of the growth curve (right panel). Results correspond to the mean of three biological replicates performed on different transformants. Error bars represent SD. Asterisks (***) indicate a p-value < 0.01 by the Tukey’s HSD test. **(D)** Immunoblots showing the expression of caspase-1 (upper panel) and caspase-8 (lower panel) in yeast lysates of cells bearing the same plasmids as in **(B)** after 5 h induction in SG medium. Membranes were hybridized with anti-caspase-1 and anti–caspase-8 antibodies. Anti-G6PDH antibody was used as loading control. A representative blot from three different experiments with different transformants is shown.

Caspases are a family of cysteine proteases that cleave their targets after aspartic acid residues, playing an essential role in inflammation, apoptosis, cell death, and development (1). Mammalian caspases are classified into two major groups: pro-inflammatory and pro-apoptotic caspases. They are produced as zymogens that are activated by proteolysis upon diverse stimuli. Among them, caspase-1 and caspase-8 are two of the most deeply characterized members. Caspase-1 exerts its function as a pro-inflammatory caspase by promoting interleukin IL-1β activation and release *via* pyroptosis, a form of regulated cell death (RCD) (2–4). Caspase-8 takes part in apoptotic RCD as an initiator caspase, upstream effector caspases in the extrinsic pathway (5). Although they intervene in different signaling hubs, they share many structural features. Both caspases are composed of a Death-fold Domain (DD: CARD—CAspase Recruitment Domain—for caspase-1; and DEDs—Death Effector Domain—for caspase-8), a long, and a short catalytic subunit (**Figure 1A**) (6).

Under specific stimuli, caspase-1 and caspase-8 are recruited to macromolecular structures, known as supramolecular-organizing centers (SMOCs), through heterotypic interactions between their DDs and the corresponding adaptors (7, 8). Next, caspases dimerize and autoactivate by proteolysis. The first cleavage between the long and short catalytic subunits leads to an increase of caspase-proteolytic activity. The second cleavage, between the long subunit and the DD, releases the caspase from the SMOC and restricts its activity. Thus, active caspases transmit the signal downstream to their substrates by proteolysis. The particular SMOC to which caspase-1 and caspase-8 are recruited, together with target specificity, accounts for the functional divergence of these two proteins (9–11). Caspase-1 and caspase-8 are promiscuous enzymes, and they do not recognize a strict target sequence motif. Rather, the conformational structure of the target might be more relevant for recognition than the sequence flanking the Asp residue (11–13). Despite their importance in human disease, especially in inflammatory diseases and cancer, respective to each class of caspases, the complex nature of their regulation and activity is not yet fully elucidated. Heterologous expression in genetically tractable experimental models could help in their characterization.

For the last 40 years, the yeast *Saccharomyces cerevisiae* has proven to be useful as a model for the functional study of human proteins and signaling pathways, partly as a consequence of the development of suitable heterologous expression tools (14). Previous reports have shown that heterologous expression of human initiator pro-apoptotic caspase-8 and caspase-10 is toxic to *S. cerevisiae*. On the contrary, executioner pro-apoptotic caspases are not toxic, unless co-expressed with an initiator caspase or expressed in their truncated active form (15–19), because they need to be activated *via* proteolysis (6). However, there is no information available regarding the expression of pro-inflammatory caspases in yeast. These caspases deserve special attention because they are crucial in defense against pathogens, cancer, auto-immune diseases, and sepsis as part of the innate immunity (20).

In this study, we heterologously express human caspase-1 in yeast for the first time to our knowledge, and compare its effects with those of caspase-8. We demonstrate that caspase-1 auto-activates and becomes toxic in yeast when overexpressed because of its own proteolytic activity. Moreover, it is able to efficiently cleave its natural substrate gasdermin D (GSDMD). This model might provide a novel platform to readily assess the function of human caspase-1 and the molecular mechanisms that lead to pyroptosis in higher cells in an easily manageable *in vivo* experimental setting.

## Materials and Methods

### Strains, Media, and Growth Conditions

The BY4741 *S. cerevisiae* strain (*MATα his3Δ1 leu2Δ0 met15Δ0 ura3Δ0*) or its *trp1::kanMX4* derivative (a gift from Ángela Sellers-Moya, Complutense University of Madrid, Madrid, Spain) were used in all experiments unless otherwise stated. DLY35 [*MATα his3Δ1 leu2Δ0 ura3Δ0 lys2Δ0 SEC7-GFP(S65T)::KanMX*] strain (a gift from Mara C. Duncan, University of North Carolina, NC, USA) (21) was used to visualize the trans-Golgi network and MVY04 strain (isogenic to BY4741, *VPH1-GFP-URA3*) to visualize vacuolar membrane. MVY04 strain was obtained by digesting de plasmid ZJOM153 (Addgene #268960) with *Nhe*I and *Stu*I and integrating the resulting *VPH1-GFP-URA3* fragment in the BY4741 strain. BY4741 *bnr1Δ*, BY4741 *bni1Δ*, BY4741 *yca1Δ*, BY4741 *nuc1Δ*, BY4741 *aif1Δ*, and BY4741 *dnm1Δ* strains were obtained from the WGD collection (Euroscarf). BGY12 (*MATα; his3-11, 15; ura3-52; leu2-3,112; ade2-1; trp1-1; psi+; ssd-; GAL+)* and BGY3240 (Isogenic BGY12, *bni1Δ::TRP*) strains were a gift from Bruce L. Goode, University of Brandeis, MA, USA. BY4741 *Bni1-GFP-HIS3MX6* strain was obtained from the Yeast-GFP Clone Collection from UCSF. The *Escherichia coli* DH5α strain was used for routine molecular biology techniques.

Synthetic dextrose (SD) medium contained 2% glucose, 0.17% yeast nitrogen base without amino acids, 0.5% ammonium sulfate and 0.12% synthetic amino acid drop-out mixture, lacking appropriate amino acids and nucleic acid bases to maintain selection for plasmids. For synthetic galactose (SG) and synthetic raffinose (SR) media, glucose was replaced with 2% (w/v) galactose or 1.5% (w/v) raffinose, respectively. All the media components were autoclaved together. *GAL1*-driven protein induction in liquid medium was performed by growing cells in SR to mid-exponential phase and then refreshing the cultures to an OD_600_ of 0.3 directly with SG lacking the appropriate amino acids to maintain selection for plasmids for 5 h. Yeast strains were incubated at 30°C.

For *GAL1* promoter modulation experiments, *GAL1*-driven protein induction in liquid medium and growth assays were performed as described above but instead of SG media, synthetic media containing different proportions of galactose and glucose was used. The final concentration of sugars was always 2% (w/v). In this case, the sugars were prepared at a 10× concentration and autoclaved separately from the other medium components.

### Plasmids

Transformation of *E. coli* and *S. cerevisiae* and other basic molecular biology methods were carried out using standard procedures. *CASP1* and *CASP1 ΔCARD* genes were amplified by standard PCR from pCl-caspase-1 (a gift of Jonathan Kagan, Boston Children’s Hospital, MA, USA) using primers CASP1_Fw, CASP1(CARD)_Fw and CASP1_Rv, respectively, all designed with *attB* flanking sites. *CASP1 D5N* uncleavable mutant was amplified by standard PCR from pLEX 307-FLAG-CASP1 D5N (a gift from Daniel A. Bachovchin, Memorial Sloan Kettering Cancer Center, NY, USA) (22) using the same primers as for *CASP1*. *CASP8* and *CASP8 ΔDED* genes were amplified by standard PCR from pcDNA3-CASP8 (a gift from Faustino Mollinedo, CIB-CSIC, Madrid, Spain) using primers CASP8_Fw, CASP8(DED)_Fw and CASP8_Rv, respectively, all designed with *attB* flanking sites. GSDMD gene was amplified from pEGFP-N1-GSDMD (a gift from Jonathan Kagan, Boston Children’s Hospital, MA, USA) using primers GSDMD_Fw and GSDMD_Rv2, both designed with *attB* flanking sites. See **Table 1** for primer sequences. The *attB*-flanked PCR products were cloned into pDONR221 vector by BP Gateway reaction (Invitrogen™) to generate entry clones. Subsequently, the inserts from the entry clones were subcloned into pAG413GAL-ccdB, pAG416GAL-ccdB, and pAG416-GAL-ccdB-EGFP vectors (Addgene kit #1000000011) (23) by LR Gateway reaction (Invitrogen™), generating the plasmids pGA413-caspase-1, pAG413-caspase-8, pAG416-caspase-1, pAG416-caspase-8, pAG413-caspase-1 ΔCARD, pAG413-caspase-8 ΔDED, pAG413-caspase-1 D5N, and pAG416-GSDMD-EGFP.

**Table 1:** Oligonucleotides used in this work.

Name	Sequence
CASP1_Fw	5′-GGGGACAAGTTTGTACAAAAAAGCAGGCTTCACCATGGCCGACAAGGTCCTG-3′
CASP1(CARD)_Fw	5′-GGGGACAAGTTTGTACAAAAAAGCAGGCTTCACCATGAACCCAGCTATGCCCAC3′
CASP1_Rv	5′-GGGACCACTTTGTACAAGAAAGCTGGGTTTTAATGTCCTGGGAAGAGGTAG-3′
CASP8_Fw	5′-GGGGACAAGTTTGTACAAAAAAGCAGGCTTCACCATGGACTTCAGCAGAAATC-3′
CASP8(DED)_Fw	5′-GGGGACAAGTTTGTACAAAAAAGCAGGCTTCACCATGAGTGAATCACAGACTTTGG-3′
CASP8_Rv	5′-GGGGACCACTTTGTACAAGAAAGCTGGGTTTTAATCAGAAGGGAAGACAAG-3′
CASP1(C285A)_Fw	5′-CATCCAGGCCGCCCGTGGTGACAGCCCTG-3′
CASP1(C285A)_Rv	5′-CTGTCACCACGGGCGGCCTGGATGATGATCAC-3′
CASP8(C360A)_Fw	5′-GTGTTTTTTATTCAGGCTGCTCAGGGGGATAACTACCAG-3′
CASP8(C360A)_Rv	5′-GTAGTTATCCCCCTGAGCAGCCTGAATAAAAAACACTTTGG-3′
GSDMD_FW	5′-GGGGACAAGTTTGTACAAAAAAGCAGGCTTCACCATGGGGTCGGCCTTTGAG-3′
GSDMD_RV2	5′-GGGGACCACTTTGTACAAGAAAGCTGGGTTGTGGGGCTCCTGGCTCAG-3′
GSDMD(D275N)_Fw	5′-CTTCCTGACAAATGGGGTCCCTGCGGAG-3′
GSDMD(D275N)_Rv	5′-GGGACCCCATTTGTCAGGAAGTTGTGGAGG-3′

Caspase-1 C285A and caspase-8 C360A catalytically inactive mutants, and GSDMD D275N uncleavable mutant were obtained by site-directed mutagenesis performed on their respective entry clone, using primers CASP1(C285A)_Fw and CASP1(C285A)_Rv primers for caspase-1 C285A; CASP8(C360A)_Fw and CASP8(C360A)_Rv primers for caspase-8 C360A; and GSDMD(D275N)_Fw and GSDMD(D275N)_Rv for GSDMD D275N. Primers are listed in **Table 1**. Subsequently, the inserts from the entry clones were subcloned into pAG413GAL-ccdB, pAG416GAL-ccdB, and pAG416GAL-ccdB-EGFP plasmids by LR Gateway reaction, generating the plasmids pAG413-caspase-1 C285A, pAG413-caspase-8 C360A, pAG416-caspase-1 C285A, pAG416-caspase-8 C360A, and pAG416-GSDMD D275N-EGFP.

The mitochondrial marker Ilv6-mCherry, encoded in the plasmid YEplac112-Ilv6-mCherry, has previously been described (24). The ER marker Sec63-mRFP, encoded in the plasmid pSM1959 (pRS425-Sec63-mRFP), was obtained from Addgene (#41837). Bni1, encoded in the plasmid PB1025 (*CEN; URA3)*, was a gift from David S. Pellman, Dana-Farber Cancer Institute, MA, USA (25).

### Western Blotting Assays

Western blotting assays were carried out by standard techniques. Cells were harvested by centrifugation and disrupted by bead beating with a FastPrep 24 (MP Biomedicals) in 50 mM Tris-HCl pH 7.5 containing 10% glycerol, 0.1% NP-40, 1% Triton X-100, 0.1% sodium dodecyl sulfate (SDS), 150 mM NaCl, 5 mM EDTA, 50 mM NaF, 50 mM glycerol phosphate, 5 mM Na_2_P_2_O_7_, 1 mM sodium orthovanadate, 3 mM Phenylmethylsulfonyl fluoride (PMSF), and Pierce Protease Inhibitor (ThermoFisher). Lysates were cleared by centrifugation at 4°C and protein concentrations were determined by measuring the OD_280_. Proteins were resolved by sodium dodecyl sulfate polyacrylamide gel electrophoresis (SDS-PAGE) in 10% acrylamide gels for caspase-1 blots and 12% for caspase-8 blots, and transferred onto nitrocellulose membranes 1 h at 110 V. For experiments with Bni1-GFP, cells were harvested by centrifugation and disrupted with 1.85 M NaOH 7,4% β-mercaptoethanol for 10 min and trichloroacetic acid (TCA) 50% for 10 min. Cells were washed with acetone twice and resuspended in 2% SDS sample buffer. Proteins were resolved by SDS-PAGE in a 7.5% acrylamide gels and transferred onto nitrocellulose membranes overnight at 30 V. Rabbit anti–caspase-1 (D7F10) antibody (Cell Signaling Technology; 1:1000 dilution), mouse anti–caspase-8 (1C12) (Cell Signaling Technology; 1:1000 dilution), and mouse anti-GFP (JL8) (Living colors, 1:1000 dilution) were used as primary antibodies to detect the expression of caspase-1, caspase-8 and proteins fused to GFP, respectively. Rabbit anti-G6PDH antibody (Sigma; 1:50000 dilution) was used as a loading control. Anti-rabbit IgG-IRDye 800CW, anti-rabbit IgG-IRDye 680LT, anti-mouse IgG-IRDye 800CW, anti-mouse IG-IRDye 680LT (all from LI-COR; at 1:5000 dilution) were used as secondary antibodies. Oddissey infrared imaging system (LI-COR) was used for developing the immunoblots.

### Flow Cytometry

Cells were cultured as previously stated. After 5 h of galactose induction, 1 ml of cell culture was harvested and incubated at 30°C with 5 µg/ml Rd 123 for 30 min in aerobic conditions, or 0.0005% PI for 2 min. For Annexin V/PI staining, we used ApoAlert™ Annexin V-FITC Apoptosis Kit. 3 ml of cell culture was harvested, washed once with protoplast buffer (0.5 mM MgCl_2_, 40 mM KPO_4_, 1.2 M sorbitol, pH 6.5), and resuspended in this same buffer supplemented with 100T zymolyase (MP Biomedicals) at a final concentration of 0.5 mg/ml. Cells were incubated for 30 min in the roller for cell wall digestion, then harvested, washed once with binding buffer 1× supplemented with 1.2 M sorbitol, resuspended in 200 µl of the same buffer and stained with 5 µl of annexin V and 10 µl of PI for 15 min.

Cells were analyzed using a FACScan (Becton Dickinson) flow cytometer through a 488-nm excitation laser and a 530/30 BP emission filter (FL1) for Rd 123 and Annexin V; and a 585/42 BP emission filter (FL2) for PI. At least 10,000 cells were analyzed for each experiment. Data were processed using FlowJo software (FlowJo LLC, Ashland, OR, USA).

### Spot Growth Assays

Spot growth assays on plates were performed by incubating transformants overnight in SR media, adjusting the culture to an OD_600_ of 0.5 and spotting samples in four serial 10-fold dilutions onto the surface of SD or SG plates lacking the appropriate amino acids to maintain selection for plasmids, followed by incubation at 30°C for 2 to 3 days.

### Cell Viability Assay

Cells were cultured as previously stated. After 5 h of galactose induction, cell viability was measured by the microcolonies method (26). Cells suspensions (5  μl) at an adjusted OD_600_ of 0.2 were poured on a thin layer of yeast peptone dextrose (YPD) agar on a microscope slide. A coverslip was placed over the samples and after 12 to 24 h viable and nonviable cells were identified based on their ability to form microcolonies.

### Microscopy Techniques

For *in vivo* bright differential interference contrast (DIC) microscopy or fluorescence microscopy, cells were cultured as previously stated, harvested by centrifugation 3000 rpm 3 min and viewed directly on the microscope. Cells were examined with an Eclipse TE2000U microscope (Nikon) using the appropriate sets of filters. Digital images were acquired with an Orca C4742-95-12ER charge-coupled device camera (Hamamatsu) and were processed with the HCImage software (Hamamatsu, Japan).

For confocal microscopy, cells were cultured as previously stated, harvested by centrifugation, and fixed with a 4% p-formaldehyde 3.4% sucrose solution for 15 min at room temperature. Then cells were washed and resuspended in phosphate buffered saline (PBS). Coverslips were treated with 5 µl of Concanavalin A (Sigma) and dried at room temperature. Adhesion of cells was performed by adding 70 µl of fixed cells over Concanavalin A treated coverslips and incubating for 30 min. ProLong™ Glass Antifade Mountant (ThermoFisher)/Glycerol (1:1) was used to avoid photobleaching. Cells were examined with an Olympus Ix83 inverted microscope, coupled to Olympus FV1200 confocal system, using the appropriate set of filters.

Observation of actin in yeast cells with rhodamine-conjugated phalloidin (Sigma) was performed as previously described (27). For FM4-64 vital staining (*N*-[3-triethylammoniumpropyl]-4-[*p*-diethylaminophenylhexatrienyl] pyridinium dibromide; Invitrogene), cells were cultured as previously stated, harvested by centrifugation and resuspended in synthetic medium. Cells were labeled with 2.4 µM FM4-64, incubated for 1.5 h at 30°C with shaking, washed in PBS and observed by fluorescence microscopy. Images were analyzed using Image J and Adobe Photoshop.

### Statistical Analysis

Data were analyzed using R. All data sets were tested for normality using the Shapiro-Wilkinson test. When a normal distribution was confirmed, a Student’s *t*-test was used for statistical comparison between two groups and one-way ANOVA test with a post-hoc Tukey’s HSD test between multiple groups. For data sets that did not show normality, a Kruskal-Wallis test with a post-hoc Dunn’s test was applied. The asterisks (*, **, ***) in the figures correspond to a p-value of <0.05, <0.01, and <0.001, respectively. Experiments were performed as biological triplicates on different clones and data with error bars are represented as mean ± standard deviation.

## Results and Discussion

### Expression of Human Caspase-1, Like Caspase-8, Inhibits Yeast Cell Growth

Pro-inflammatory caspase-1 and pro-apoptotic caspase-8 share a similar domain architecture and distribution of proteolytic sites for autoactivation (**Figure 1A**). To study whether pro-inflammatory caspase-1 might exert toxic effects on the yeast cell, as previously reported for initiator caspase-8 (16, 18, 19), we cloned the cDNAs encoding for both human caspases in the same expression vector under the control of galactose-inducible *GAL1* promoter. *S. cerevisiae* cells expressing each of these caspases failed to grow on solid galactose-containing media (**Figure 1B**) and significantly decelerated growth in liquid cultures (**Figure 1C**). The doubling time calculated through the growth curve raised from 2.5 h for control cells to 4.5 h for cells expressing caspase-1 and almost 10 h for cells expressing caspase-8. Biomass after 24 h of culture in galactose-based liquid medium was reduced by two-fold for caspase 1- and by 6-fold by caspase-8 as compared to the empty vector control (**Figure 1C**). Our results suggest that both human caspases become active in our model by sheer overproduction in the absence of further stimuli, probably because they self-interact bypassing the requirement for nucleating factors. This supports the proximity-driven dimerization model proposed for the pro-apoptotic initiator caspases (28), which would also extend to pro-inflammatory caspase-1. The severe toxicity of caspase-8 in yeast is consistent with previous reports (16, 18, 19). The relatively milder toxicity of caspase-1 observed in liquid culture could be due either to a lower intrinsic activity or autoactivation ability, or to a differential specificity on essential heterologous protein targets in yeast.

### Caspase-1 and Caspase-8 Are Self-Processed in Yeast

The activation of executioner pro-apoptotic caspases and pro-inflammatory caspases requires dimerization and autoproteolysis of the pro-caspase at the cleavage sites that link the long and short catalytic subunits (6). By Western blotting analysis, we demonstrated that both caspases were efficiently expressed and self-processed in yeast into their predicted active forms, as we were able to detect protein bands corresponding to the p33 and p20 cleaved subunits for caspase-1, and the p41 and p18 cleaved subunits for caspase-8 (**Figure 1D**). Thus, expression of caspase-1 in *S. cerevisiae*, like that of caspase-8, leads to its auto-processing and activation.

### Caspase-1 and Caspase-8 Protease Activity and Caspase-1 Autoprocessing Are Essential for Their Toxicity in Yeast

**Figure 2:**
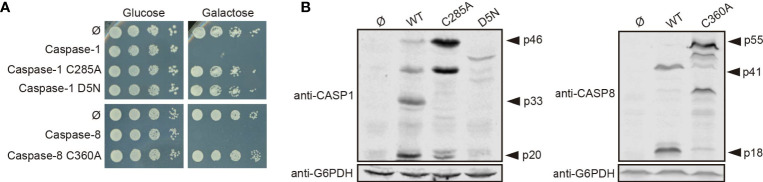
Caspase-1 and caspase-8 toxicity is a consequence of their proteolytic activity. **(A)** Yeast spot assay performed as in **Figure 1B** but using BY4741 strain bearing pAG413-caspase-1, pAG413-caspase-8, or plasmids bearing their respective catalytically inactive mutants pAG413-caspase-1 C285A and pAG413-caspase-8 C360A, and the uncleavable mutant pAG413-caspase-1 D5N. **(B)** Immunoblots showing the expression in yeast lysates of cells bearing the same plasmids as in panel **(A)** after 5 h of induction in SG medium. Membranes were hybridized with anti–caspase-1, and anti–caspase-8 antibodies. Anti-G6PDH antibody was used as a loading control. Representative assays from three different experiments with distinct transformants are shown in all cases.

To learn whether the toxic effect caused in yeast cells by both caspases reflected their protease activity, we generated a catalytically inactive mutant for each caspase by site-directed mutagenesis, in which the cysteine residues located at their respective active centers were replaced by alanine (caspase-1 C285A and caspase-8 C360A). As expected, these mutant proteins neither impaired yeast growth (**Figure 2A**) nor could we detect significant amounts of their proteolyzed subunits by Western blotting on yeast lysates (**Figure 2B**). Thus, we conclude that caspase proteolytic activity is necessary for caspase processing, autoactivation, and toxicity in yeast. Besides, we cloned in the same vector the uncleavable caspase-1 D5N mutant, in which the five Asp residues that allow caspase-1 autoprocessing are mutated to Asn, an amino acid that is not targeted by this protease (22). As with catalytically inactive caspase-1, yeast growth was not impaired by expression of the D5N mutant (**Figure 2A**) and we could not detect caspase-1 proteolyzed subunits (**Figure 2B**), emphasizing that autoprocessing of caspase-1 is also necessary for its activation and toxicity. However, we observed lower expression levels of the D5N mutant version as compared with wild type caspase-1, suggesting that these mutations affect protein stability, which could also contribute to the lack of toxicity observed.

### Caspase-1 Causes Cell Death in *S. cerevisiae*, but not Through Apoptosis

**Figure 3:**
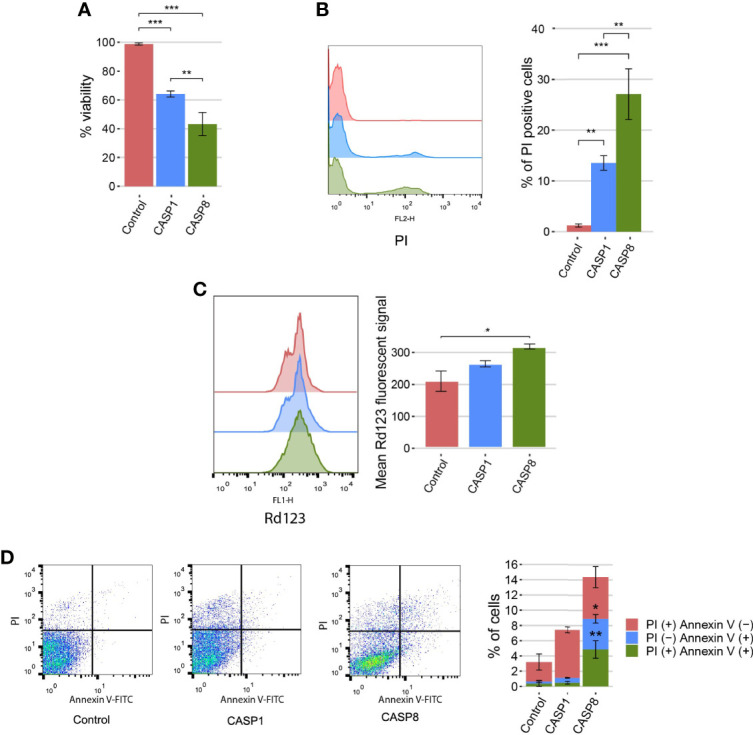
Caspase-1 and caspase-8 cause reduction in cell viability, cell death, and mitochondrial membrane hyperpolarization. **(A)** Graph showing the percentage of viable cells determined by a cell viability assay of BY4741 strain bearing the same plasmids as in **Figure 1B** after 5 h of induction in SG medium. **(B)** Stacked histograms (n=10,000) showing PI fluorescent signal by flow cytometry in abscissae (left panel) and graph showing the percentage of positive PI-stained cells of each population (right panel) of BY4741 strain bearing the same plasmids as in panel **(A)**. **(C)** Stacked histograms (n=10,000) showing Rd123 fluorescent signal by flow cytometry in abscissae (left panel) and graph showing the mean Rd123 fluorescent signal of each population (right panel) of BY4741 strain bearing the same plasmids as in panel **(A)**. **(D)** Dot plots (left panels) and stacked bar graph (right panel) showing the percentage of PI (+) annexin V (−), PI (−) annexin V (+), and PI (+) annexin V (+) cells of BY4741 strain bearing the same plasmids as in panel **(A)**. Only data from caspase-1 and caspase-8 were considered for comparison in this case. Results correspond to the mean of three biological replicates performed on different clones in all cases. Error bars represent SD. Asterisks (*, **, ***) indicate a *p* value < 0.05 by Dunn’s test in panel **(A)**, and a p-value < 0.01 and 0.001, respectively, by the Tukey’s HSD test in panels **(B, C)**. In panel **(D)**, asterisks (*, **) represent a p-value < 0.05 and 0.01, respectively, by Student’s T-test.

The cell death phenotype induced by pro-apoptotic initiator caspases in yeast is characterized by reactive oxygen species (ROS) production, a decrease in cell viability, and propidium iodide (PI) uptake. Mechanistically, cells show some features of apoptosis, such as phosphatidylserine externalization, but cell death does not rely on yeast apoptotic machinery (16, 19). To gain a better insight into the caspase-1 terminal phenotype, as compared with that of caspase-8, we evaluated whether growth inhibition was accompanied by a reduction in cell viability and cell death under the same conditions. Cell viability was measured based on the ability of yeasts to form microcolonies, and we observed a significant decrease in cell viability for both caspases (**Figure 3A**). In addition, cell death was analyzed by flow cytometry using PI as an indicator of loss of plasma membrane selective permeability. Likewise, we found a statistically significant differential increase in cell death for both caspases (**Figure 3B**). Then, we measured mitochondrial membrane potential by staining caspase-1– and caspase-8–expressing cells with rhodamine 123 (Rd123), and analyzing them by flow cytometry. To avoid misinterpretation of the data as a consequence of the presence of dead cells, we co-stained cells with PI, and only the population of PI-negative cells was taken into consideration (**Figure S1A**). No differences in the basal fluorescent signal emitted by non-stained control cells were observed (**Figure S1B**). We detected a slight increase in the mitochondrial membrane potential as the mean fluorescent signal of Rd123 increased for both caspase-1– and caspase-8–expressing cells, which was statistically significant for the latter (**Figure 3C**).

Transient mitochondrial hyperpolarization can be considered a marker for apoptosis in yeast. To further characterize the cell death routine induced by caspases, we obtained yeast protoplasts after caspase-1 and caspase-8 expression and stained cells with annexin V to monitor phosphatidylserine externalization, as an apoptotic marker, and PI to assess plasma membrane integrity. It is generally accepted that PI (+) Annexin V (−) and PI (−) Annexin V (+) populations correspond to primary necrotic and apoptotic cells, respectively; and PI (+) Annexin V (+) are late apoptotic or secondary necrotic cells (29). To validate our methodology, we treated cells with increasing concentrations of acetic acid and determined the percentage of cells that belonged to each population for the concentrations tested. As previously described (30), low doses of acetic acid primarily induced cell death by apoptosis, as PI (−) Annexin V (+) and PI (+) Annexin V (+) predominated in these samples. On the contrary, at higher doses, the percentage of cells undergoing primary necrosis [PI (+) Annexin V (−)] increased, whereas the percentage of apoptotic cells did not suffer major changes (**Figure S2A**). As for heterologously expressed caspases, there were significant differences between both of them. Although caspase-1 mainly induced cell death by necrosis [PI (+) annexin V (−)], caspase-8–expressing cells died from apoptosis [PI (−) annexin (+)], necrosis [PI (+) Annexin V (−)], or secondary necrosis [PI (+) annexin (+)] (**Figure 3D**). Furthermore, we assessed caspase-1 and caspase-8 toxicities in yeast mutant strains lacking key components involved in yeast apoptosis (i.e. Yca1, Nuc1, Aif1, and Dnm1) (31). Neither caspase-1 nor caspase-8 toxicity depended on any of these proteins (**Figure S2B**), reflecting that the induction of apoptosis-like events in yeast is not the primary cause of cell death. Taken together, these results indicate that the expression of caspase-1 and caspase-8 leads to a decrease in cell viability, involving cell death and modest mitochondrial membrane hyperpolarization. Furthermore, consistent with our results above, caspase-8 effects are stronger in all cases. Besides, caspase-8, but not caspase-1, induces yeast apoptosis to a certain extent, as detected by annexin V staining, although this cell death subroutine is not the main cause for growth inhibition.

### Expression of Caspase-1 and Caspase-8 Differentially Affects Organelle Morphology in Yeast

**Figure 4:**
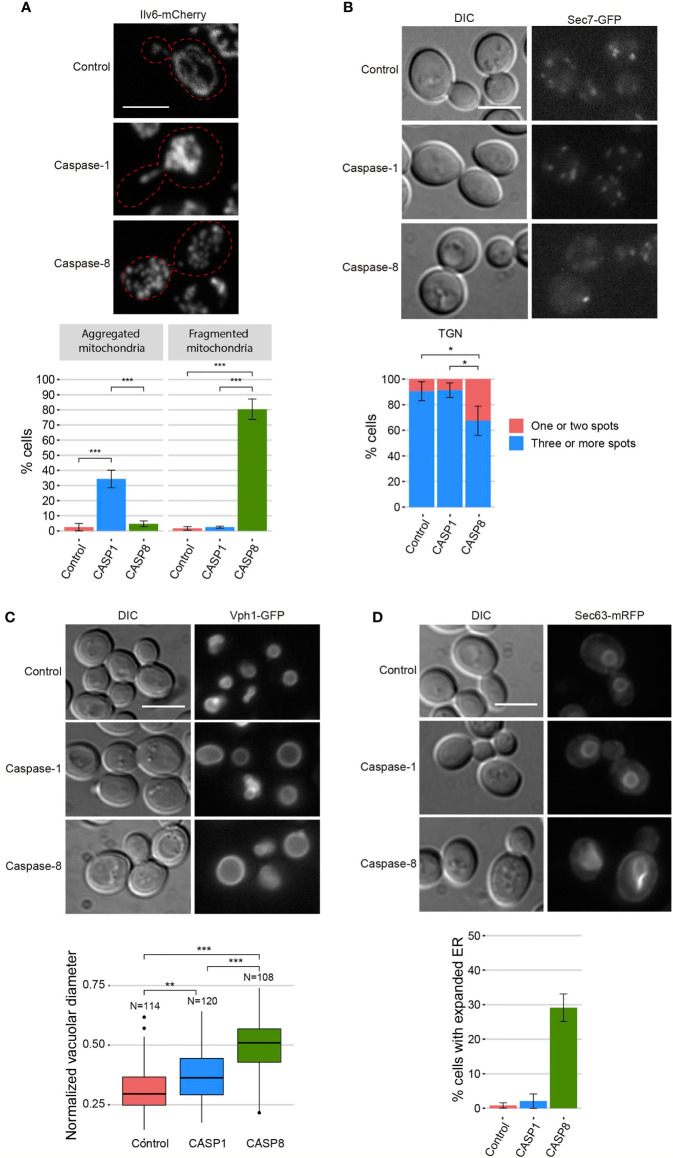
Caspase-1 and caspase-8 overexpression alters subcellular organelles. **(A)** Confocal fluorescent microscopy and quantification (n > 100) of the mitochondrial phenotype of BY4741 *trp1Δ* strain bearing the mitochondrial marker pYEp-lac112-Ilv6-Cherry and the same plasmids as in **Figure 1B**. **(B)** Fluorescent and bright field differential interferential contrast (DIC) microscopy (left panel) and quantification (n > 100) of the number of TGN spots per cell (right panel) of DLY35 strain, bearing the TGN marker Sec7-GFP, and the same plasmids as in **Figure 1B**. **(C)** Fluorescent and bright field (DIC) microscopy (upper panel) and boxplot (n > 100) representing the vacuolar diameter normalized by cell diameter (lower panel) of MVY04 strain, bearing the vacuolar marker Vph1-GFP, and the same plasmids as in **Figure 1B**. **(D)** Fluorescent and bright field (DIC) microscopy (upper panel) and quantification (n > 100) of the percentage of cells showing expanded ER (lower panel) of BY4741 strain bearing the ER marker pRS425-Sec63-mRFP and the same plasmids as in **Figure 1B**. Abnormal ER expansions were considered following previously described criteria (32). Caspase-1 and caspase-8 expression were induced in SG medium for 5 h in all cases. All scale bars indicate 5 µm. Results correspond to the mean of three biological replicates performed on different transformants. Error bars represent SD. Asterisks (*, **, ***) indicate a p-value < 0.05 by the Tukey’s HSD test in panels **(A, B)** and < 0.01 and 0.001, respectively, by the Dunn’s test in panel **(C)**.

We investigated the putative damages that these caspases might be causing to cellular organelles. First, to visualize mitochondrial organization, we co-expressed each caspase with an Ilv6-mCherry fusion as a mitochondrial marker. The mitochondrial tubular network was disrupted in both cases, although there were some differences between the two caspases. While mitochondria from cells expressing caspase-1 formed large aggregates in 35% of the cells, those from cells expressing caspase-8 were fragmented in 80% of the cells (**Figure 4A**). Second, we assessed trans-Golgi network (TGN) integrity by expressing caspases in a strain marked with the TGN protein Sec7-GFP. Caspase-1 expression did not affect TGN, whereas caspase-8 caused the aggregation of Golgi cisternae into one or two big spots in around 30% of the cells (**Figure 4B**). Third, we studied vacuole morphology by expressing each caspase in a strain tagged with the vacuolar protein Vph1-GFP. Both caspases caused an increment of the vacuolar diameter, although the effect was more prominent in the case of caspase-8 (**Figure 4C**). Finally, we evaluated the endoplasmic reticulum (ER) structure by co-expressing each caspase with Sec63-mRFP as an ER marker. In this case, no differences with the control were perceived for caspase-1–expressing cells, whereas 30% of caspase-8–expressing cells displayed an expanded ER (**Figure 4D**). Previous studies report that ER expansion might be a consequence of increased ER membrane biogenesis to adapt to different stress signals (32). In sum, the expression of both caspases in yeast leads to significant organellar alterations, but the effect of caspase-8 is more severe than that of caspase-1, especially at the ER and Golgi levels.

### The Yeast Actin Cytoskeleton Is Altered by Expression of Human Caspases

**Figure 5:**
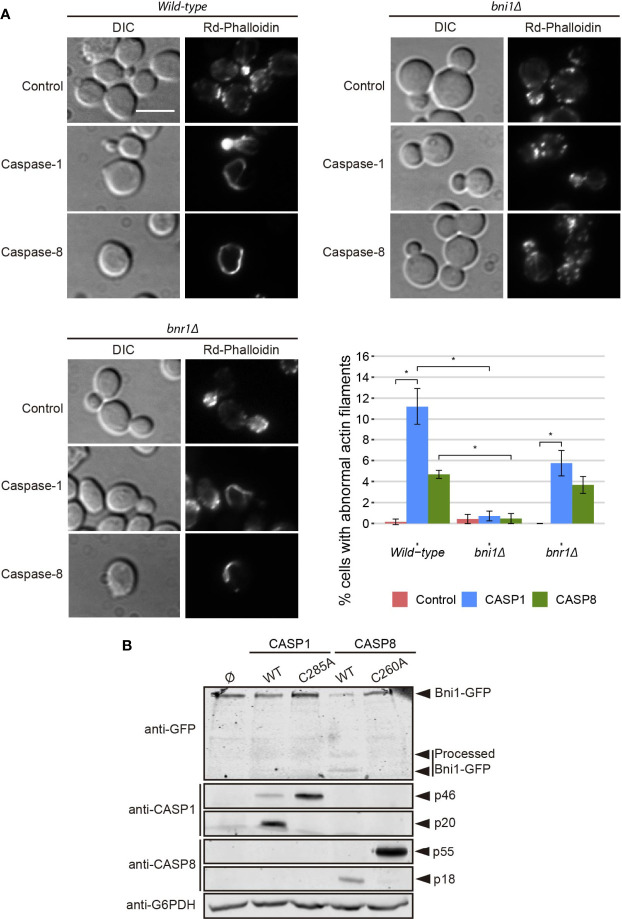
Caspase-1 and caspase-8 overexpression alters actin cytoskeleton *via* Bni1 proteolysis. **(A)** Acting staining with rhodamine-phalloidin, bright field (DIC) microscopy, and quantification (n > 200) of the percentage of cells showing abnormal actin structures of BY4741 wild type, and isogenic *bni1Δ* and *bnr1Δ* strains bearing the same plasmids as in **Figure 1B**. Scale bar indicates 5 µm. Error bars represent SD. Asterisks (*) indicate a p-value < 0.05 by the Dunn’s test. **(B)** Immunoblots showing the degradation of Bni1-GFP and the expression of caspase-1, caspase-8,and their correspondent protease-death mutants in yeast lysates of BY4741 *Bni1-GFP-HIS2MX6* strain bearing pAG416-Caspase-1, pAG416-Caspase-1 C285A, pAG416-Caspase-8, and pAG416-Caspase-8. pAG416 empty vector (Ø) was used as a negative control. Membranes were hybridized with anti-GFP, anti–caspase-1, and anti–caspase-8 antibodies. Anti-G6PDH antibody was used as a loading control. Caspase-1 and caspase-8 expression was induced in SG medium for 5 h in all cases. A representative blot from three different experiments with distinct transformants is shown.

As shown above, the dramatic growth defect observed in heterologous caspase-expressing yeast cells was accompanied by a modest loss of plasma membrane permeability, as determined by vital PI staining. This is consistent with budding arrest rather than cell lysis. However, microscopic observations did not hint at a specific cell cycle arrest pattern in these cultures (data not shown), so we investigated the impact of caspases expression on the actin cytoskeleton, as it drives essential polarized secretion to promote and maintain budding. Actin staining with rhodamine-conjugated phalloidin revealed that instead of polarized patches at the growing bud, about 11% of the cells expressing caspase-1 and 5% of the cells expressing caspase-8 displayed abnormal long thick actin cytoplasmic structures, which were never observed in control cultures (**Figure 5A**). These observations suggest that growth arrest in caspase-expressing yeast cells could be at least partially attributed to dysfunction of the actin cytoskeleton.

Formins are actin-nucleating proteins that function by promoting and regulating the assembly of the actin cytoskeleton in eukaryotic cells (33). Previous studies reported that loss of the N-terminal domain of the yeast formin Bni1 led to the formation of long cytoplasmic actin filamentous structures, resembling the ones here induced by caspases, because of the uncontrolled actin polymerization caused by dysregulation of this protein (34, 35). We hypothesized that the observed phenotype could be a consequence of Bni1 proteolysis by caspase-1 and -8 in a region proximal to the N-terminal domain and that, in such case, the formation of these structures should be prevented in a *bni1Δ* strain. Therefore, we stained the actin cytoskeleton in caspase-expressing yeast cells individually deleted for the genes encoding each of the formins (Bni1 and Bnr1). We could still observe the formation of these abnormal actin structures in the *bnr1Δ* but there was a significant decrease in the *bni1Δ* background (**Figure 5A**), supporting our postulate that Bni1 cleavage is responsible for this phenomenon. Then, we used a plasmid-expressing Bni1 from its own promoter to complement Bni1 function in the *bni1Δ* genetic background and, as expected, the appearance of abnormal actin structures upon caspase expression was restored (**Figure S3A**). This experiment was reproduced in a strain derived from the W303 genetic background with similar results (**Figure S3B**).

To further confirm this hypothesis, we co-expressed a Bni1-GFP fusion with each caspase and analyzed yeast lysates by Western blotting with anti-GFP antibodies. We detected that both caspases degraded Bni1, particularly caspase-8, as the levels of Bni1-GFP decreased and some degradation bands—absent in control lysates—appeared. These changes could not be detected when the respective protease-dead mutant caspases were expressed (**Figure 5B**). This implies that Bni1 is a probable direct substrate of caspase-1 and -8 in yeast and that the collapse of the actin cytoskeleton into these abnormal filaments as a consequence of Bni1 cleavage likely contributes to yeast growth arrest. To our knowledge, no previous works have described any direct substrates of human caspases in yeast. Disruption of the mitochondrial network, TGN or vacuoles are rather non-specific damages and could account for many different causes. However, the formation of these aberrant actin structures is more specific and could be an interesting tool to assess caspase activity in yeast by microscopy or immunoblot.

### Auto-Processing of Caspase-1, but not Caspase-8, Can Be Modulated by Adjusting Expression Levels

**Figure 6:**
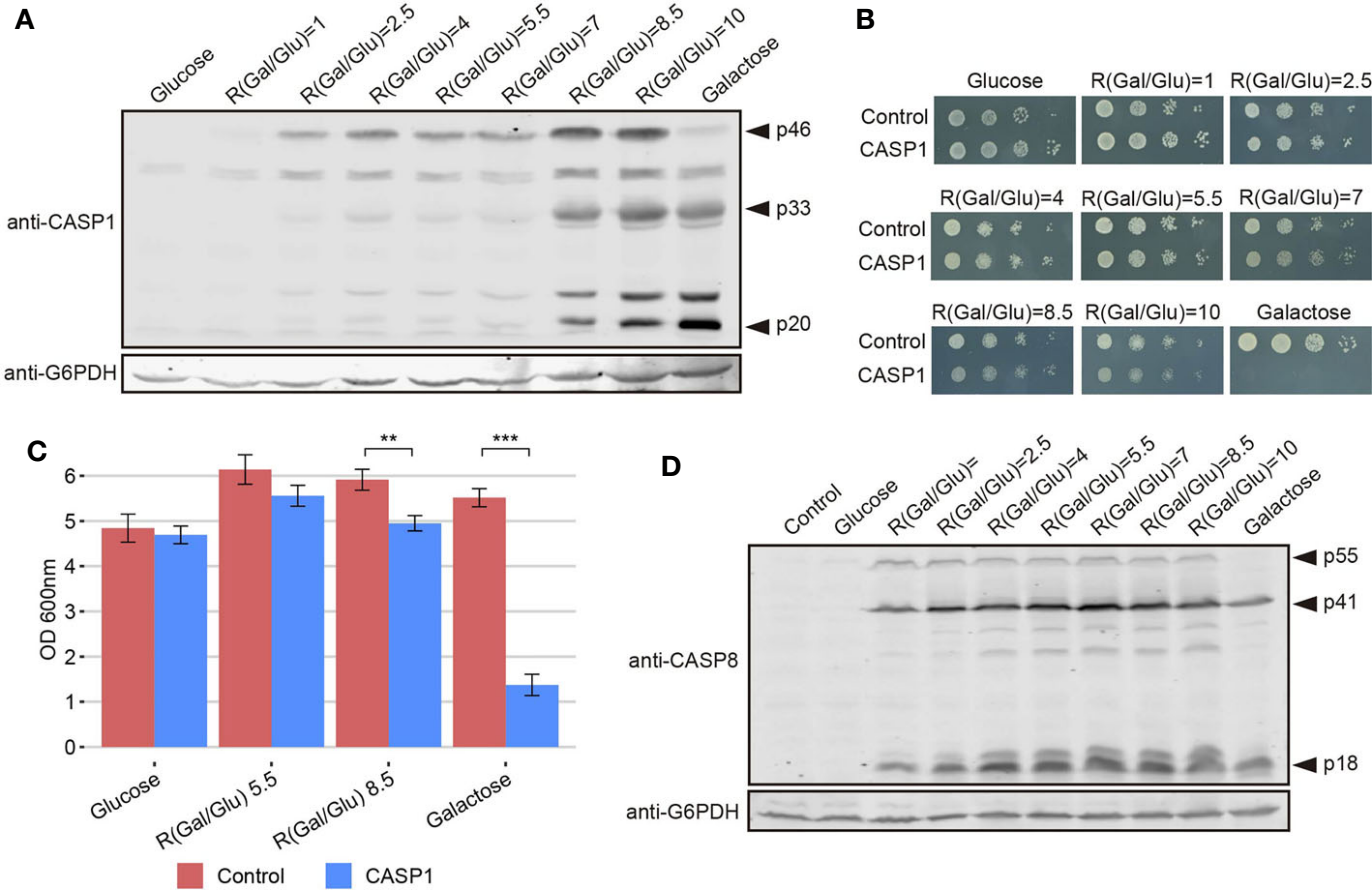
The modulation of caspase-1 expression, but not of caspase-8, under the *GAL1* promoter limits its auto-processing and toxicity. **(A)** Immunoblot showing the expression of caspase-1 in yeast lysates of BY4741 strain bearing pAG413-caspase-1. Cells were cultured in synthetic media containing the indicated Gal/Glu ratios with a final concentration of sugars of 2% for 5 h. Cells cultured in SD medium were used as a negative control of expression and cells cultured in SG media as a positive control. Membrane was hybridized with anti-caspase-1 antibody. Anti-G6PDH antibody was used as a loading control. A representative assay from two different experiments with distinct transformants is shown. **(B)** Spot growth assay performed with the same strain and the same ratios of galactose/glucose as in panel **(A)**, but in solid media. Cells bearing pAG413 empty plasmid were used as growth control. A representative assay from three different experiments with distinct transformants is shown. **(C)** Measurement of OD_600_ after 24 h of incubation in media containing a R(Gal/Glu) = 5.5 and R(Gal/Glu) = 8.5 of BY4741 strain bearing either pAG413-caspase-1 or the pAG13 empty plasmid as growth control. As in panel **(B)**, cells cultured in SD medium were used as a negative control of expression, and cells cultured in SG media as a positive control of expression. Results correspond to the mean of three biological replicates performed on different transformants. Error bars represent SD. Asterisks (**, ***) indicate a p-value < 0.01 and 0.001, respectively, by the Student’s T-test. **(D)** Immunoblotting performed as in panel **(A)** but with BY4741 strain bearing pAG413-caspase-8 or the pAG413 empty plasmid as control. Membrane was hybridized with anti–caspase-8 antibody. Anti-G6PDH antibody was used as a loading control. A representative assay from two different experiments with different transformants is shown.

The development of yeast-based models for the study of human pathways should be more versatile if finely tuned regulatory mechanisms can be reproduced in the heterologous model. Thus, although a growth inhibition readout may be optimal for devising pharmacologic or genetic screens, in putative Synthetic Biology settings, high toxicity can be acaveat. In our model, the overexpression of caspase-1 results presumably in its dimerization and auto-processing, thus leading to toxicity. However, we show above that its effects are not as dramatic as those caused by pro-apoptotic caspase-8, likely because of a less efficient activation by auto-cleavage. We hypothesized that restricting caspase-1 expression levels could prevent its dimerization and consequently reduce its toxicity in yeast. It has been reported that *GAL1* promoter induction depends on the ratio between galactose and glucose available in the culture medium, which determines galactose uptake, rather than on the total amount of galactose. It was suggested that the competitive binding of these sugars to hexose transporters is responsible for this phenomenon (36). Thus, we cultured caspase-1 transformants in media containing different galactose/glucose ratios ranging from 1 to 10, always preserving a final concentration of sugars of 2%, and after 5 h of induction we analyzed caspase-1 expression by Western blotting. As shown in **Figure 6A**, we confirmed that not only the level of expression of this protein but also its relative auto-processing capability increased gradually as the Gal/Glu ratio augmented. At those ratios for which pro-caspase-1 was detectable but the signal for its p33 and p20 subunits was weak compared to control with galactose, caspase activity should be low. To test whether this modulation of caspase-1 expression and autoprocessing correlated with a reduction in toxicity, we performed a spot assay using the same sugar ratios in growth media (**Figure 6B**). Yeast growth was observed in all Gal/Glu ratios. However, in the higher ratios (8.5 and 10) we detected a reduction in the size of colonies that reflects that caspase-1 was being processed to sufficient active form. To confirm that threshold concentrations for autoprocessing did not dramatically compromise viability, we chose R(Gal/Glu)=5.5 and R(Gal/Glu)=8.5 among the different ratios tested and analyzed yeast growth in liquid media after 24 h of induction. The 5.5 Gal/Glu ratio allowed us to express an inactive caspase-1 (p33 and p20 bands were barely detectable in cell extracts) and the 8.5 Glu/Gal ratio led to an active caspase-1 (p33 and p20 bands indicated the presence of the active protein). Like in solid media, although we observed a statistically significant decrease in OD_600_ due to caspase-1 expression in both the control with galactose alone and R(Gal/Glu)=8.5, toxicity was highly reduced in the latter condition (**Figure 6C**). Thus, this experimental setting should provide a sensitive platform for evaluating caspase-1 activity in viable yeast cells in the future.

In contrast, we could not modulate caspase-8 activity under the same conditions tested for caspase-1 (**Figure 6D**) or even at lower Gal/Glu ratios ranging from 0.25 to 4 (**Figure S4**). For R(Gal/Glu) ≤ 1, we could not detect caspase-8 expression over the control in glucose, and for R(Gal/Glu) ≥ 2.5 caspase-8 was already autoactivated.

### Deletion of Caspase-1 CARD and Caspase-8 DED Domains Have Opposite Effects

**Figure 7:**
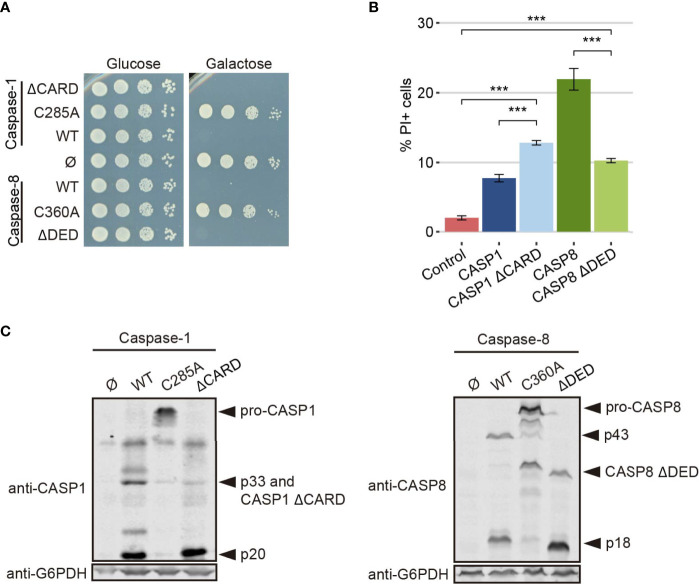
CARD and DED domains have opposite effects on the respective caspase activity. **(A)** Spot growth assay of BY4741 strain bearing pAG413-caspase-1 and pAG413-caspase-8, the plasmids with their respective catalytically inactive mutants pAG413-caspase-1 C285A and pAG413-caspase-8 C360A, and the plasmids with their respective mutant lacking of DD pAG413-caspase-1 ΔCARD and pAG413-caspase-8 ΔDED. pAG413 empty vector was used as a negative control. Cells were cultured on SD (glucose) or SG (galactose) agar media for induction of caspase-1 and caspase-8 expression. A representative assay from three different experiments with different transformants is shown. **(B)** Graph showing the percentage of positive PI stained cells of each population of BY4741 strain bearing the same plasmids as in panel **(A)** after 5 h of induction in SG medium. Results correspond to the mean of three biological replicates performed on different transformants. Error bars represent SD. Asterisks (***) indicate a p-value < 0.001 by the Tukey’s HSD Test. Only significance levels between mutant caspases (CASP1 ΔCARD and CASP8 ΔDED, their wild type counterpart, and control cells are represented. **(C)** Immunoblots showing the expression of wild type and the different mutants of caspase-1 (left panel) and caspase-8 (right panel) in yeast lysates of the cells bearing the same plasmids as in **(A)** after 5 h induction in SG medium. Membranes were hybridized with anti–caspase-1 and anti–caspase-8 antibodies. Anti-G6PDH antibody was used as a loading control. A representative assay from three different experiments with different transformants is displayed.

To test the strength of our model in detecting changes in caspase activity, we produced a truncated version of each caspase lacking its DD (caspase-1 ΔCARD and caspase-8 ΔDED, respectively). These domains facilitate the recruitment and dimerization of caspases (5, 37, 38), so we expected that their deletion would reduce protease-dependent toxicity. Contrary to these expectations, the truncated versions of these proteins were as toxic as their wild type counterparts in a spot assay under *GAL1*-inducing conditions, as shown in **Figure 7A**, indicating that DD-mediated dimerization is not a strict prerequisite for their activation. However, when we performed PI staining after 5 h of induction in a galactose-containing medium and analyzed cells by flow cytometry, we observed opposite effects for each truncated caspase. The elimination of the CARD domain in caspase-1 increased the percentage of PI+ cells compared to the full length caspase, while deletion of DED domain for caspase-8 decreased the percentage of cells that lost selective permeability (**Figure 7B**). We then checked whether these mutants were subject to auto-cleavage in yeast like the wild type proteins. Indeed, we could detect by immunoblot of cell lysates the p20 subunit for caspase-1 ΔCARD and the p18 subunit for caspase-8 ΔDED (**Figure 7C**).

**Figure 8:**
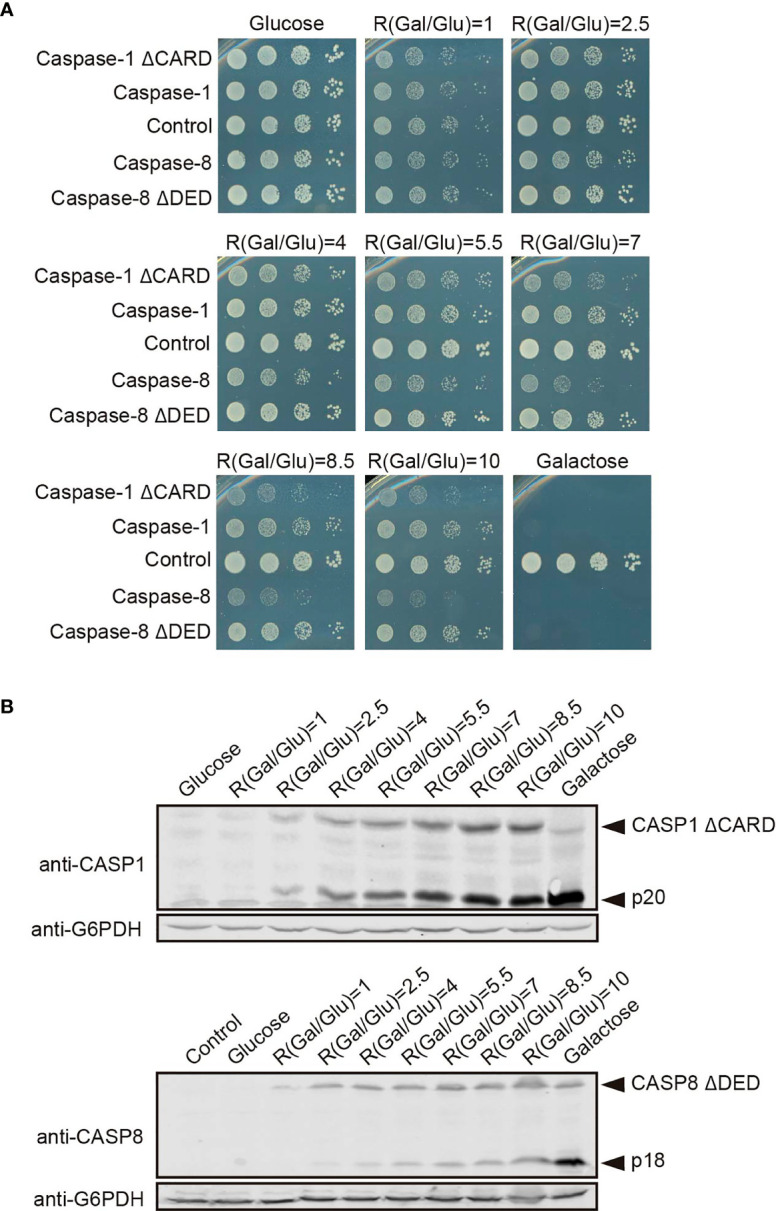
The modulation of Caspase-1 ΔCARD and Caspase-8 ΔDED expression under the *GAL1* promoter confirms their opposite effect on caspase activity. **(A)** Spot growth assay carried out as in **Figure 6B** but with BY4741 strain bearing pAG413-caspase-1 and pAG413-caspase-8, or plasmids with their respective mutant lacking DD pAG413-caspase-1 ΔCARD and pAG413-caspase-8 ΔDED. pAG413 empty vector was used as a negative control. A representative assay from three different experiments with distinct transformants is shown. **(B)** Immunoblotting carried out as in **Figure 6A** but with BY4741 strain bearing pAG413-caspase-1 ΔCARD (upper panel) and pAG413-caspase-8 ΔDED (lower panel). pAG413 empty vector was used as a negative control. Membranes were hybridized with anti-caspase-1 and anti-caspase-8 antibody. Anti-G6PDH antibody was used as a loading control. A representative assay from two different experiments with different transformants is shown.

Next, we used the approach described in the previous section for modulating the *GAL1* promoter to verify the increment of caspase-1 activity in the absence of its CARD domain, and the decrease of caspase-8 activity in the absence of its DED domain. In a spot assay using ratios of galactose/glucose ranging from 1 to 10, we could observe a gradual increase of toxicity for each caspase (**Figure 8A**). Consistent with PI staining, cell growth impairment and decrease in colony size appeared at lower ratios –corresponding to lower expression levels of the corresponding protein—for caspase-1 ΔCARD than the full-length protein, and at higher ratios for caspase-8 ΔDED mutant than for the corresponding full-length caspase. Next we performed Western blotting from cells expressing caspase-1 ΔCARD and caspase-8 ΔDED, following an analogous strategy to the one described for caspase-1 and caspase-8 in **Figures 6A, D**. As shown in **Figure 8B**, caspase-1 ΔCARD p20 cleaved subunit could be detected at very low ratios of Gal/Glu and gradually increased as the percentage of galactose raised, while this subunit could only be detected at high ratios of Gal/Glu for the full-length protein (see **Figure 6A**). On the contrary, the cleaved p18 subunit from caspase-8 ΔDED was detected in low levels in lysates from the increasing Gal/Glu ratios as compared to the galactose alone sample.

These results may reflect that, although DDs facilitate caspase dimerization and activation under physiological conditions by bringing closer caspase monomers to their corresponding SMOCs, at higher expression levels caspase monomers may interact with each other through other regions of the protein. Indeed, in our model the CARD domain restrains caspase-1 activity. The proximity-driven dimerization model is compatible with the induced conformation model (28), which argues that the interaction of caspases with the SMOCs ends up in their activation because it triggers a conformational change. In this sense, CARD orientation under basal conditions may prevent caspase-1 activation, and the interaction with its SMOC, the inflammasome, elicits a conformational change within this domain that allows caspase-1 activation. The overexpression of a truncated version of caspase-1 without CARD could bypass this need for a conformational change, consequently leading to a higher caspase-1 activity. However, removal of DED reduced caspase-8 toxicity, suggesting these DD might play different roles depending on the caspase. The physiological relevance of these results needs to be assessed in a higher eukaryote model. In human cells, caspases must be tightly regulated to preserve cell integrity, but the proteins involved in their control are probably missing in *S. cerevisiae*, as it lacks pathways closely related to those in which caspase-1 or -8 are involved. Our model provides a neat platform in which we can assess *in vivo* the intrinsic activity of caspases in the absence of other layers of regulation.

### Expression of Caspase-1 in Yeast Can Be Used as a Platform to Characterize Caspase-1 Substrates

**Figure 9:**
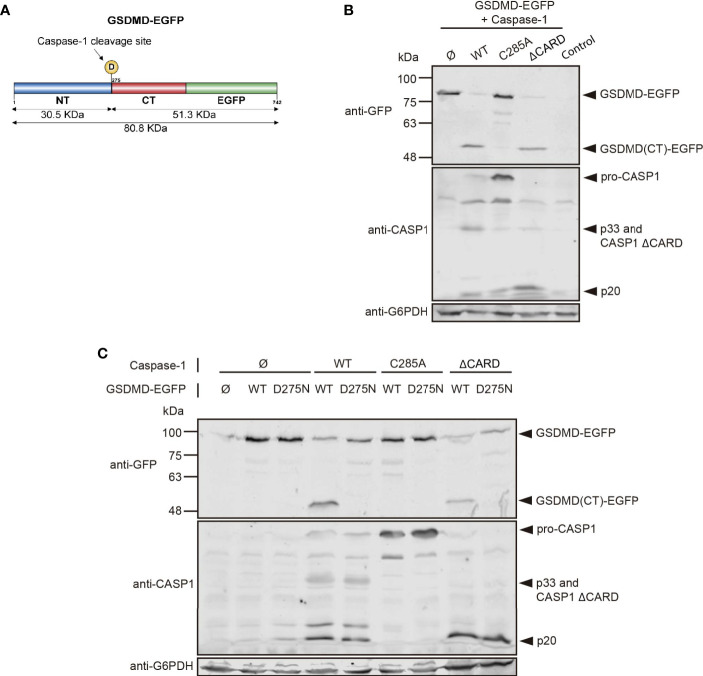
Caspase-1 cleaves its substrate GSDMD in yeast. **(A)** Schematic representation of GSDMD depicting its N-terminal domain (NT; blue) and C-terminal domain (CT; red), fused in C-terminal to EGFP (green). The D275 residue susceptible of caspase-1 cleavage and the molecular weight of the cleavage products are indicated. **(B)** Immunoblot showing GSDMD-EGFP processing in yeast lysates of cells bearing pAG416-GSDMD-EGFP and pAG413-caspase-1 WT, C285A, or ΔCARD, respectively, after 5 h induction in SG medium. pAG413 and pAG416-EGFP plasmids were used as controls. **(C)** Immunoblot showing GSDMD-EGFP processing in yeast lysates of cells bearing pAG416-GSDMD-EGFP WT or D275N and pAG413-caspase-1 WT, C285A, or ΔCARD, respectively, after 5 h induction in SG medium. pAG413 and pAG416-EGFP plasmids were used as controls. Membranes were hybridized with anti-caspase-1 and anti-GFP antibodies. Anti-G6PDH antibody was used as a loading control. A representative blot from three different experiments with different transformant clones is shown.

Caspase-1 main best-known substrates in mammalian cells are IL1, IL-8, and gasdermin D (GSDMD). This last one is the main effector of pyroptosis and allows the release of mature interleukins because cleavage by caspase-1 at D275 releases its N-terminal domain, which forms pores in the plasma membrane (3, 39). To assess whether caspase-1 expressed in yeast showed activity on its native substrates, we co-expressed it with GSDMD fused to EGFP (**Figure 9A**), both under *GAL1* promoter, and evaluated GSDMD cleavage by Western blotting. As shown in **Figure 9B**, GSDMD-EGFP expressed itself correctly, with an expected size of ≈75-85 KDa. Caspase-1 and its mutant ΔCARD were able to degrade GSDMD, generating a fragment of approximately 50-55 kDa, which corresponds to the molecular weight expected for the GSDMD C-terminal domain fused to EGFP after GSDMD N-terminal domain release. We could not detect major differences in GSDMD degradation between WT and ΔCARD caspase-1, despite the increased toxicity displayed by the latter. Thus, under our experimental conditions, the CARD domain does not seem to be implicated in the binding and cleavage of GSDMD by caspase-1. As expected, protease-dead caspase-1 did not induce GSDMD degradation. To verify whether caspase-1 was processing GSDMD at D275 and maintained its specificity towards this substrate, we constructed a GSDMD D275N uncleavable mutant. The degradation band that we had observed previously when co-expressing WT GSDMD and either caspase-1 or caspase-1 ΔCARD was no longer detectable when the D275N mutated form of GSDMD was co-expressed (**Figure 9C**). This confirms that autoactivated caspase-1 in yeast can process its native substrates specifically when they are co-expressed, resulting in an alternative tool for exploring its targets and the contribution of specific aspartate residues as susceptible targets for caspase-1 cleavage.

Overall, this study provides evidence that the yeast *S. cerevisiae* can be exploited as an alternative tool to study the structure and activity on substrates of pro-inflammatory caspase-1 in an easy-to-handle unicellular *in vivo* model, as previously reported for caspase-8. This setting may be useful for the design of platforms for the performance of molecular, pharmacologic or genetic screens in the near future.

## Data Availability Statement

The original contributions presented in the study are included in the article/**Supplementary Material**. Further inquiries can be directed to the corresponding authors.

## Author Contributions

MV performed the experiments and wrote the manuscript. MM and VJC revised the manuscript, and were responsible for conceptualization, project management and resources. All authors contributed to the article and approved the submitted version.

## Funding

This research is possible thanks to funding from Grant PID2019-105342GB-I00 from Ministerio de Ciencia e Innovación (Spain).

## Conflict of Interest

The authors declare that the research was conducted in the absence of any commercial or financial relationships that could be construed as a potential conflict of interest.

## Abbreviations

DD, death-fold domain; CARD, caspase recruitment and activation domain; CASP1, DED, death effector domain; RCD, regulated cell death; SMOC, supra-molecular organizing center; Caspase-1; CASP8, Caspase-8; ROS, reactive oxygen species; PI, propidium iodide, Rd123, rhodamine 123; DHE, dihydroethidium; TGN, trans-Golgi network; ER, endoplasmic reticulum; GSDMD, Gasdermin D; WGD, whole genome deletion; SD, synthetic dextrose; SG, synthetic galactose; SR, synthetic raffinose; OD, optical density; SDS, sodium dodecyl sulfate; PMSF, phenylmethylsulfonyl fluoride; SDS-PAGE, sodium dodecyl sulfate polyacrylamide gel electrophoresis; TCA, trichloroacetic acid; DIC, differential interference contrast; YPD, yeast peptone dextrose; PBS, phosphate buffered saline; WT, wild type.

## References

[1] Shalini S Dorstyn L Dawar S Kumar S. Old, New and Emerging Functions of Caspases. Cell Death Differ (2015) 22:526–39. 10.1038/cdd.2014.216 PMC4356345 25526085

[2] Sollberger G Strittmatter GE Garstkiewicz M Sand J Beer HD. Caspase-1: The Inflammasome and Beyond. Innate Immun (2014) 20:115–25. 10.1177/1753425913484374 23676582

[3] Sborgi L Ruhl S Mulvihill E Pipercevic J Heilig R Stahlberg H. GSDMD Membrane Pore Formation Constitutes the Mechanism of Pyroptotic Cell Death. EMBO J (2016) 35:1766–78. 10.15252/embj.201694696 PMC5010048 27418190

[4] Ding J Wang K Liu W She Y Sun Q Shi J. Pore-Forming Activity and Structural Autoinhibition of the Gasdermin Family. Nature (2016) 535:111–6. 10.1038/nature18590 27281216

[5] Tummers B Green DR. Caspase-8: Regulating Life and Death. Immunol Rev (2017) 277:76–89. 10.1111/imr.12541 28462525 PMC5417704

[6] Ramirez MLG Salvesen GS. A Primer on Caspase Mechanisms. Semin Cell Dev Biol (2018) 82:79–85. 10.1016/j.semcdb.2018.01.002 29329946 PMC6043420

[7] Kagan JC Magupalli VG Wu H. SMOCs: Supramolecular Organizing Centres That Control Innate Immunity. Nat Rev Immunol (2014) 14:821–6. 10.1038/nri3757 PMC4373346 25359439

[8] Nanson JD Kobe B Ve T Death TIR. And RHIM: Self-Assembling Domains Involved in Innate Immunity and Cell-Death Signaling. J Leukoc Biol (2019) 105:363–75. 10.1002/JLB.MR0318-123R 30517972

[9] Chang DW Xing Z Capacio VL Peter ME Yang X. Interdimer Processing Mechanism of Procaspase-8 Activation. EMBO J (2003) 22:4132–42. 10.1093/emboj/cdg414 PMC175803 12912912

[10] Boucher D Monteleone M Coll RC Chen KW Ross CM Teo JL. Caspase-1 Self-Cleavage Is an Intrinsic Mechanism to Terminate Inflammasome Activity. J Exp Med (2018) 215:827–40. 10.1084/jem.20172222 PMC5839769 29432122

[11] Julien O Wells JA. Caspases and Their Substrates. Cell Death Differ (2017) 24:1380–9. 10.1038/cdd.2017.44 PMC5520456 28498362

[12] Timmer JC Salvesen GS. Caspase Substrates. Cell Death Differ (2007) 14:66–72. 10.1038/sj.cdd.4402059 17082814

[13] Wang K Sun Q Zhong X Zeng M Zeng H Shi X. Structural Mechanism for GSDMD Targeting by Autoprocessed Caspases in Pyroptosis. Cell (2020) 180:941–55.e20. 10.1016/j.cell.2020.02.002 32109412

[14] Laurent JM Young JH Kachroo AH Marcotte EM. Efforts to Make and Apply Humanized Yeast. Brief Funct Genomics (2016) 15:155–63. 10.1093/bfgp/elv041 PMC4803062 26462863

[15] Srinivasula SM Ahmad M MacFarlane M Luo ZW Huang ZW Fernandes-Alnemri T. Generation of Constitutively Active Recombinant Caspases-3 and -6 by Rearrangement of Their Subunits. J Biol Chem (1998) 273:10107–11. 10.1074/jbc.273.17.10107 9553057

[16] Kang JJ Schaber MD Srinivasula SM Alnemri ES Litwack G Hall DJ. Cascades of Mammalian Caspase Activation in the Yeast Saccharomyces Cerevisiae. J Biol Chem (1999) 274:3189–98. 10.1074/jbc.274.5.3189 9915859

[17] Wright ME Han DK Carter L Fields S Schwartz SM Hockenbery DM. Caspase-3 Inhibits Growth in Saccharomyces Cerevisiae Without Causing Cell Death. FEBS Lett (1999) 446:9–14. 10.1016/S0014-5793(99)00159-3 10100604

[18] Puryer MA Hawkins CJ. Human, Insect and Nematode Caspases Kill Saccharomyces Cerevisiae Independently of YCA1 and Aif1p. Apoptosis (2006) 11:509–17. 10.1007/s10495-006-5114-2 16538379

[19] Lisa-Santamaria P Neiman AM Cuesta-Marban A Mollinedo F Revuelta JL Jimenez A. Human Initiator Caspases Trigger Apoptotic and Autophagic Phenotypes in Saccharomyces Cerevisiae. Biochim Biophys Acta (2009) 1793:561–71. 10.1016/j.bbamcr.2008.12.016 PMC2647587 19166881

[20] Martinon F Mayor A Tschopp J. The Inflammasomes: Guardians of the Body. Annu Rev Immunol (2009) 27:229–65. 10.1146/annurev.immunol.021908.132715 19302040

[21] Aoh QL Graves LM Duncan MC. Glucose Regulates Clathrin Adaptors at the Trans-Golgi Network and Endosomes. Mol Biol Cell (2011) 22:3671–83. 10.1091/mbc.e11-04-0309 PMC3183021 21832155

[22] Ball DP Taabazuing CY Griswold AR Orth EL Rao SD Kotliar IB. Caspase-1 Interdomain Linker Cleavage is Required for Pyroptosis. Life Sci Alliance (2020) 3(3):e202000664. 10.26508/lsa.202000664 32051255 PMC7025033

[23] Alberti S Gitler AD Lindquist S. A Suite of Gateway Cloning Vectors for High-Throughput Genetic Analysis in Saccharomyces Cerevisiae. Yeast (2007) 24:913–9. 10.1002/yea.1502 PMC2190539 17583893

[24] Fernandez-Acero T Bertalmio E Luna S Mingo J Bravo-Plaza I Rodriguez-Escudero I. Expression of Human PTEN-L in a Yeast Heterologous Model Unveils Specific N-Terminal Motifs Controlling PTEN-L Subcellular Localization and Function. Cells (2019) 8(12):1512. 10.3390/cells8121512 PMC6952770 31779149

[25] Buttery SM Yoshida S Pellman D. Yeast Formins Bni1 and Bnr1 Utilize Different Modes of Cortical Interaction During the Assembly of Actin Cables. Mol Biol Cell (2007) 18:1826–38. 10.1091/mbc.e06-09-0820 PMC1855024 17344480

[26] Palermo V Falcone C Mazzoni C. Apoptosis and Aging in Mitochondrial Morphology Mutants of *S. cerevisiae* . Folia Microbiol (Praha) (2007) 52:479–83. 10.1007/BF02932107 18298044

[27] Jimenez J Cid VJ Cenamor R Yuste M Molero G Nombela C. Morphogenesis Beyond Cytokinetic Arrest in Saccharomyces Cerevisiae. J Cell Biol (1998) 143:1617–34. 10.1083/jcb.143.6.1617 PMC2132980 9852155

[28] Bao Q Shi Y. Apoptosome: A Platform for the Activation of Initiator Caspases. Cell Death Differ (2007) 14:56–65. 10.1038/sj.cdd.4402028 16977332

[29] Carmona-Gutierrez D Bauer MA Zimmermann A Aguilera A Austriaco N Ayscough K. Guidelines and Recommendations on Yeast Cell Death Nomenclature. Microbial Cell (Graz Austria) (2018) 5:4–31. 10.15698/mic2018.01.607 PMC5772036 29354647

[30] Ludovico P Sousa MJ Silva MT Leao CL Corte-Real M. Saccharomyces Cerevisiae Commits to a Programmed Cell Death Process in Response to Acetic Acid. Microbiol (Reading) (2001) 147:2409–15. 10.1099/00221287-147-9-2409 11535781

[31] Carmona-Gutierrez D Eisenberg T Buttner S Meisinger C Kroemer G Madeo F. Apoptosis in Yeast: Triggers, Pathways, Subroutines. Cell Death Differ (2010) 17:763–73. 10.1038/cdd.2009.219 20075938

[32] Bircham PW Papagiannidis D Lüchtenborg C Ruffini G Brügger B Schuck S. Control of endoplasmic reticulum membrane biogenesis by regulators of lipid metabolism. Bioxriv (2020). 10.1101/2020.02.23.961722

[33] Evangelista M Zigmond S Boone C. Formins: Signaling Effectors for Assembly and Polarization of Actin Filaments. J Cell Sci (2003) 116:2603–11. 10.1242/jcs.00611 12775772

[34] Pruyne D Evangelista M Yang C Bi E Zigmond S Bretscher A. Role of Formins in Actin Assembly: Nucleation and Barbed-End Association. Sci (New York NY) (2002) 297:612–5. 10.1126/science.1072309 12052901

[35] Juanes MA Piatti S. The Final Cut: Cell Polarity Meets Cytokinesis at the Bud Neck in S. cerevisiae. Cell Mol Life Sci (2016) 73:3115–36. 10.1007/s00018-016-2220-3 PMC4951512 27085703

[36] Escalante-Chong R Savir Y Carroll SM Ingraham JB Wang J Marx CJ. Galactose Metabolic Genes in Yeast Respond to a Ratio of Galactose and Glucose. Proc Natl Acad Sci USA (2015) 112:1636–41. 10.1073/pnas.1418058112 PMC4321281 25605920

[37] Boatright KM Salvesen GS. Mechanisms of Caspase Activation. Curr Opin Cell Biol (2003) 15:725–31. 10.1016/j.ceb.2003.10.009 14644197

[38] Srinivasula SM Poyet JL Razmara M Datta P Zhang Z Alnemri ES. The PYRIN-CARD Protein ASC Is an Activating Adaptor for Caspase-1. J Biol Chem (2002) 277:21119–22. 10.1074/jbc.C200179200 11967258

[39] Shi J Zhao Y Wang K Shi X Wang Y Huang H. Cleavage of GSDMD by Inflammatory Caspases Determines Pyroptotic Cell Death. Nature (2015) 526:660–5. 10.1038/nature15514 26375003

