# Supplementary material for: Heterologous Expression and Auto-Activation of Human Pro-Inflammatory Caspase-1 in Saccharomyces cerevisiae and Comparison to Caspase-8
Source: Front Immunol. 2021 Jul 14;12:668602. doi: 10.3389/fimmu.2021.668602 (PMC8317575; doi:10.3389/fimmu.2021.668602)
Supplement: Supplementary file 1 [file Presentation_1.pdf]

Supplementary Figure S1

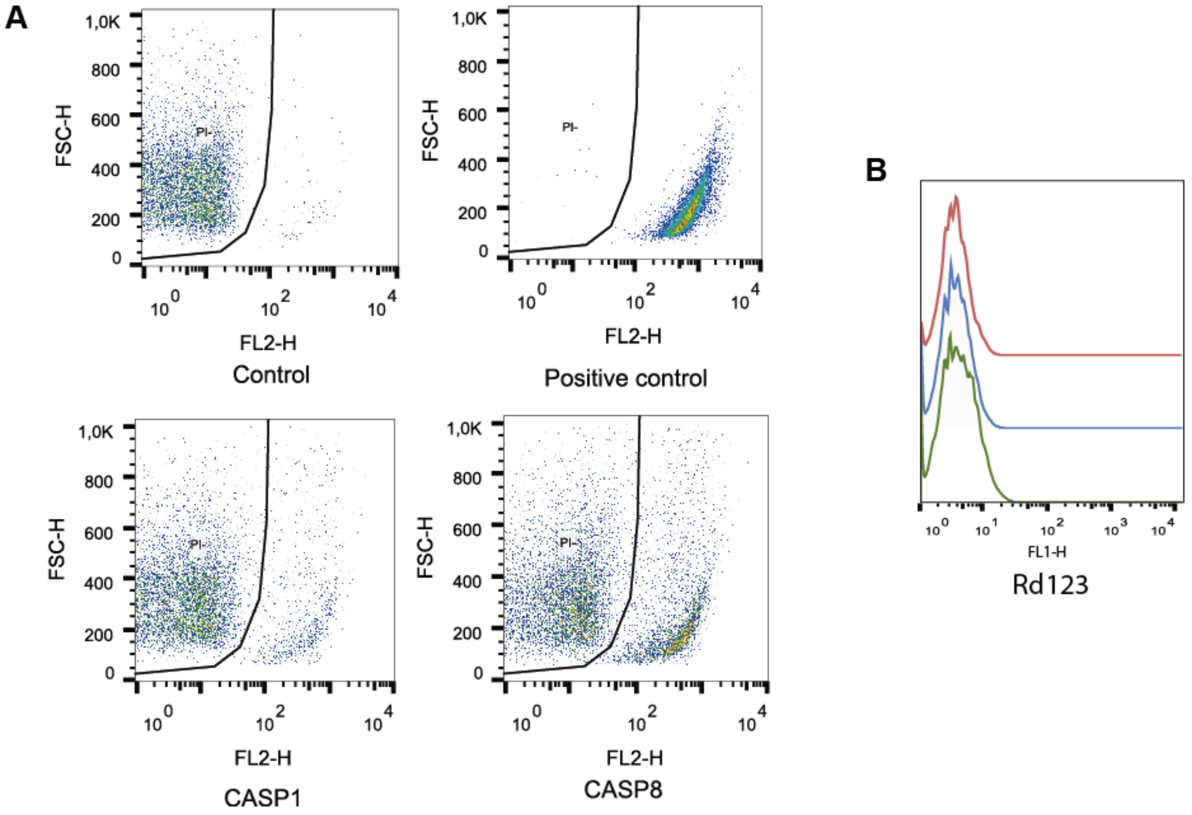

Figure S1. (A) Dot plots (n=10,000) showing PI gates from flow cytometry analysis of BY4741 strain bearing pAG413-caspase-1 and pAG413-caspase-8, after 5 h of induction in SG medium. pAG413 empty vector was used as a negative control. Boiled cells were used as a positive control. **(B)** Stacked histograms (n=10,000) showing basal fluorescent signal of cells unstained with Rd123 by flow cytometry of BY4741 strain bearing the same plasmids as in panel **(A)** after 5 h of induction in SG medium.

Supplementary Figure S2

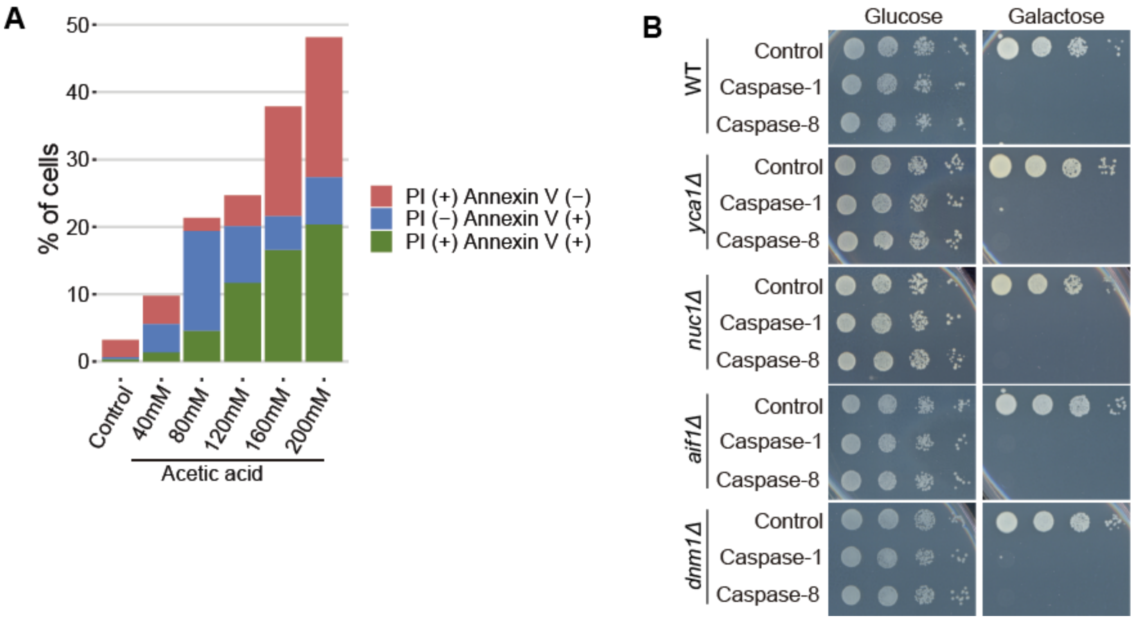

Figure S2. (A) Stacked bar graph showing the percentage of PI (+) Annexin V (-), PI (-) Annexin V (+), and PI (+) Annexin V (-) cells of BY4741 strain bearing the pAG413 empty vector untreated (control) or treated with increasing concentrations of acetic acid. **(B)** Spot growth assay of BY4741 wild type (WT), *yca1Δ*, *nuc1Δ*, *aif1Δ*, and *dnm1Δ* strains bearing pAG413-caspase-1 and pAG413-caspase-8. pAG413 empty vector was used as a negative control. Cells were cultured on SD and SG agar media for repression and induction of caspase-1 and caspase-8 expression, respectively.

Supplementary Figure S3

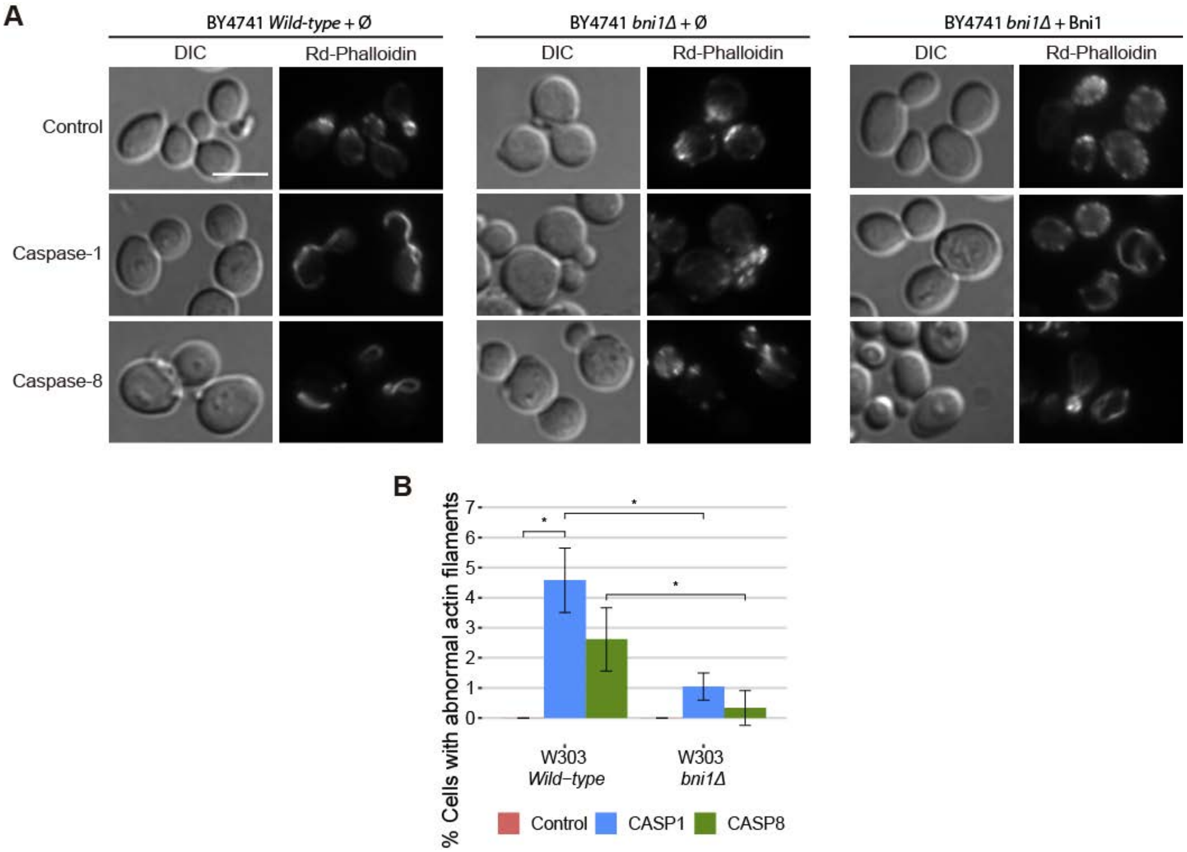

Figure S3. (A) Bright field (DIC) microscopy and acting staining with rhodamine-phalloidin of BY4741 wild type bearing pAG416 empty vector, and isogenic bni1Δ transformed with the same empty vector or PB1025 to complement Bni1 function. These strains were co-transformed with pAG413-caspase-1 and pAG413-caspase-8 plasmids. pAG413 empty plasmid was used as a negative control. Scale bar indicates 5 µm. **(B)** Quantification (n > 200) of the percentage of cells showing abnormal actin structures after rhodamine-phalloidin staining of BGY12 (wild type) and BGY3240 *(bni1Δ)* strains bearing pAG413-caspase-1 and pAG413-caspase-8. pAG413 empty vector was used as a negative control.

Supplementary Figure S4

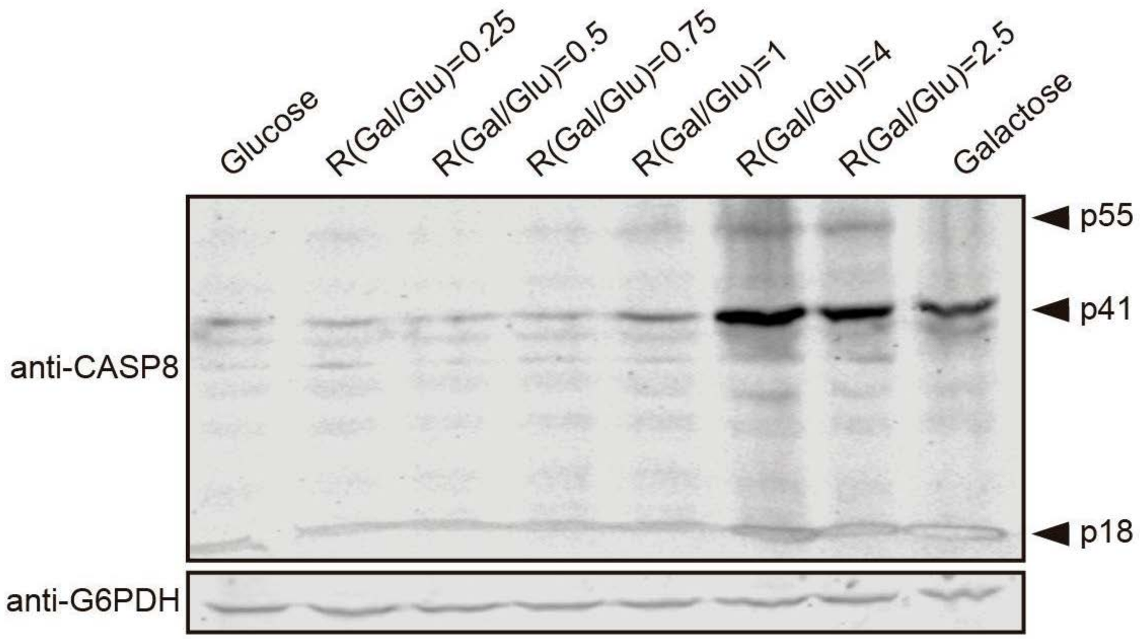

Figure S4. Immunoblot showing the expression of caspase-8 in yeast lysates of BY4741 strain bearing pAG413-caspase-8. Cells were cultured in synthetic media containing the indicated Gal/Glu ratios with a final concentration of sugars of 2% for 5 h. Cells cultured in SD medium were used as a negative control of expression and cells cultured in SG media as a positive control. Membrane was hybridized with anti-caspase-8 antibody. Anti-G6PDH antibody was used as a loading control.
